# Characterization of Definitive Regulatory B Cell Subsets by Cell Surface Phenotype, Function and Context

**DOI:** 10.3389/fimmu.2021.787464

**Published:** 2021-12-20

**Authors:** Savannah D. Neu, Bonnie N. Dittel

**Affiliations:** ^1^ Versiti Blood Research Institute, Milwaukee, WI, United States; ^2^ Department of Microbiology and Immunology, Medical College of Wisconsin, Milwaukee, WI, United States

**Keywords:** regulatory B cell (Breg cell), IL-10, Treg, autoimmunity, EAE (experimental autoimmune encephalomyelitis), multiple scleorsis (MS), BD_L_

## Abstract

Regulatory B cell or “Breg” is a broad term that represents the anti-inflammatory activity of B cells, but does not describe their individual phenotypes, specific mechanisms of regulation or relevant disease contexts. Thus, given the variety of B cell regulatory mechanisms reported in human disease and their animal models, a more thorough and comprehensive identification strategy is needed for tracking and comparing B cell subsets between research groups and in clinical settings. This review summarizes the discovery process and mechanism of action for well-defined regulatory B cell subsets with an emphasis on the mouse model of multiple sclerosis experimental autoimmune encephalomyelitis. We discuss the importance of conducting thorough B cell phenotyping along with mechanistic studies prior to defining a particular subset of B cells as Breg. Since virtually all B cell subsets can exert regulatory activity, it is timely for their definitive identification across studies.

## Introduction

Alongside T cells, B cells or B lymphocytes make up the adaptive immune system that recognizes and retains memory of foreign antigen encounters. The B cell receptor (BCR) or immunoglobulin (Ig) is a cell surface protein that recognizes intact antigens. BCR binding to cognate shapes activates B cells and leads to downstream protective functions including antigen presentation, upregulation of effector cell surface proteins and soluble factors and B cell differentiation into specialized effector subsets (1, 2). B cells are continuously produced in the bone marrow (BM) to replenish and replace expended populations in peripheral tissues (3, 4). High B cell turnover sustains a large and diverse BCR repertoire, that can protect against a vast array of foreign antigens, but can also be self-reactive (3, 5, 6). To minimize pathological autoimmunity regulatory cell subsets, which include T cells, macrophages and B cells, control and resolve inflammation and reestablish homeostasis through many mechanisms (7–10). Targeting regulatory cells for depletion to boost anti-cancer responses or expansion to dampen autoimmunity is a current therapeutic strategy (9, 11–13).

Although there are differences between the human immune system and animal models, B cells have similar functions and residence across species. B cells are divided into two broad subsets based on origin. Conventional B2 B cells in mouse and human are BM-derived and account for most B cell populations in circulation and in the secondary lymphoid organs including lymph nodes, spleen and mucosal sites (14). In mice, B1 B cells were shown to be derived from the yolk sac/fetal liver and largely self-renew in the peritoneal cavity and surrounding abdominal tissues and secrete large amounts of IgM for innate-like immune protection (15, 16). B1 B cells are subdivided into B1a and B1b in mice (17). Both B2 and B1 B cells can engage in antigen-specific immune responses, secrete protective antibodies and become further specialized based on 1) localization to tissue types/structures and proximity to other cells; 2) BCR affinity for self or foreign ligands and 3) availability of growth signals, stimulation, and differentiation factors. In both mice and humans, BM-derived B cells follow a maturation trajectory from immature states (pre-B, pro-B, transitional) to mature marginal zone and follicular subsets (18). Naïve B1 and B2 B cells become activated through their BCR and related signaling (co-stimulation, cytokines) and differentiate into long-lived memory or antibody secreting plasma cells (19, 20). During differentiation into plasma cells, activated B cells in both mice and humans, downregulate canonical B cell markers (CD19, surface Ig, Pax5) and upregulate transcriptional programs needed for efficient protein folding and secretion (IRF4, Blimp-1, XBP1) (19, 21–24). In this process, B cells mature through an intermediary plasmablast stage characterized by high proliferation and migration to effector sites (19, 21).

**Figure 1:**
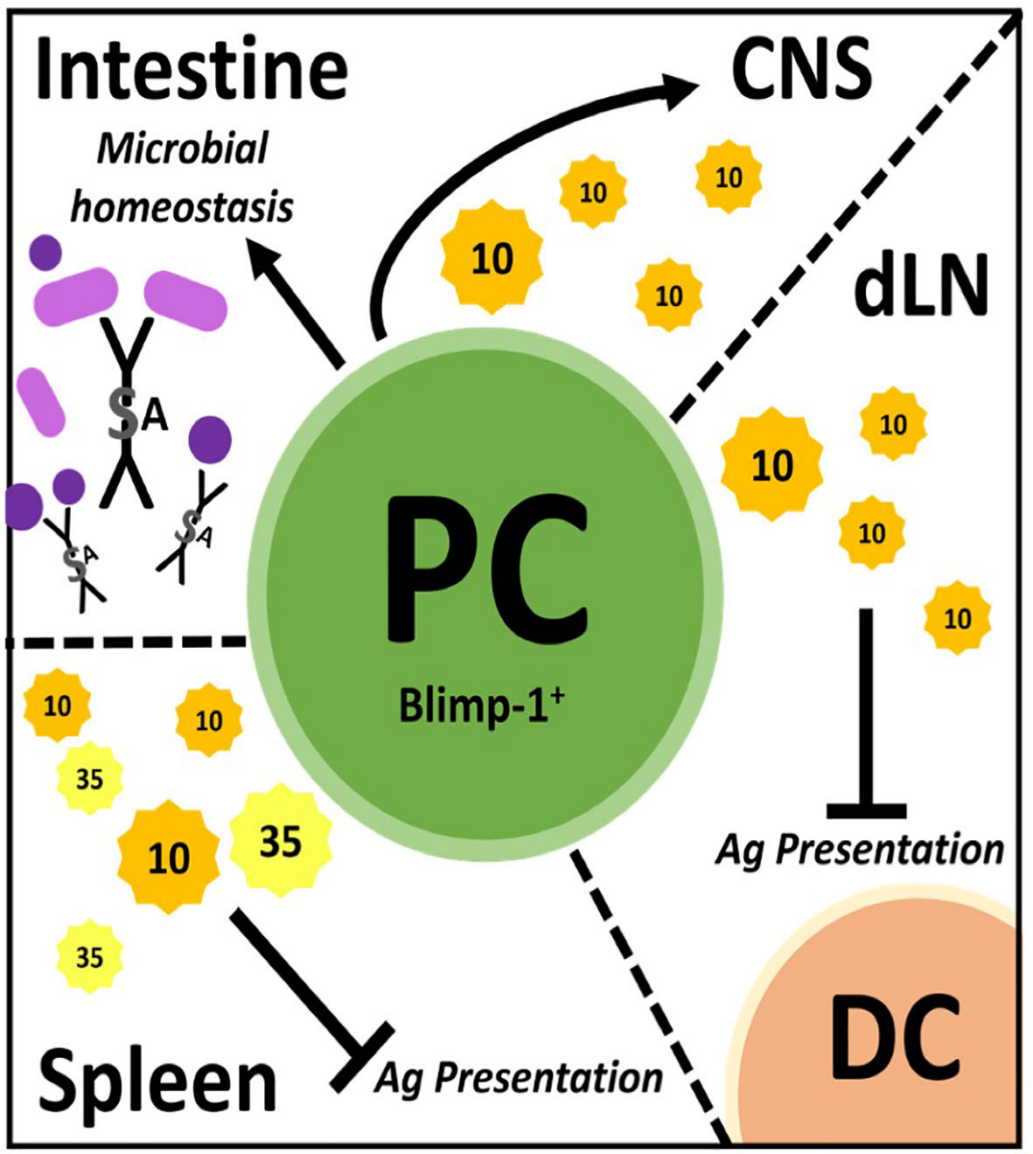
Plasmablast/plasma cells (PC) regulate through many mechanisms in different tissues. PC facilitate microbial homeostasis at mucosal surfaces like the small and large intestine through secretion of lgA, a dimerized lg with added features. These PC can migrate to the central nervous system (CNS) and secrete IL-10 to dampen inflammation therein. In draining lymph nodes (dLN), PC secrete IL-10 to inhibit antigen (Ag) presentation by dendritic cells (DC), thereby dampening immune responses. PC secrete IL-10 and IL-35 in the spleen which is believed to inhibit Ag presentation therein.

**Figure 2:**
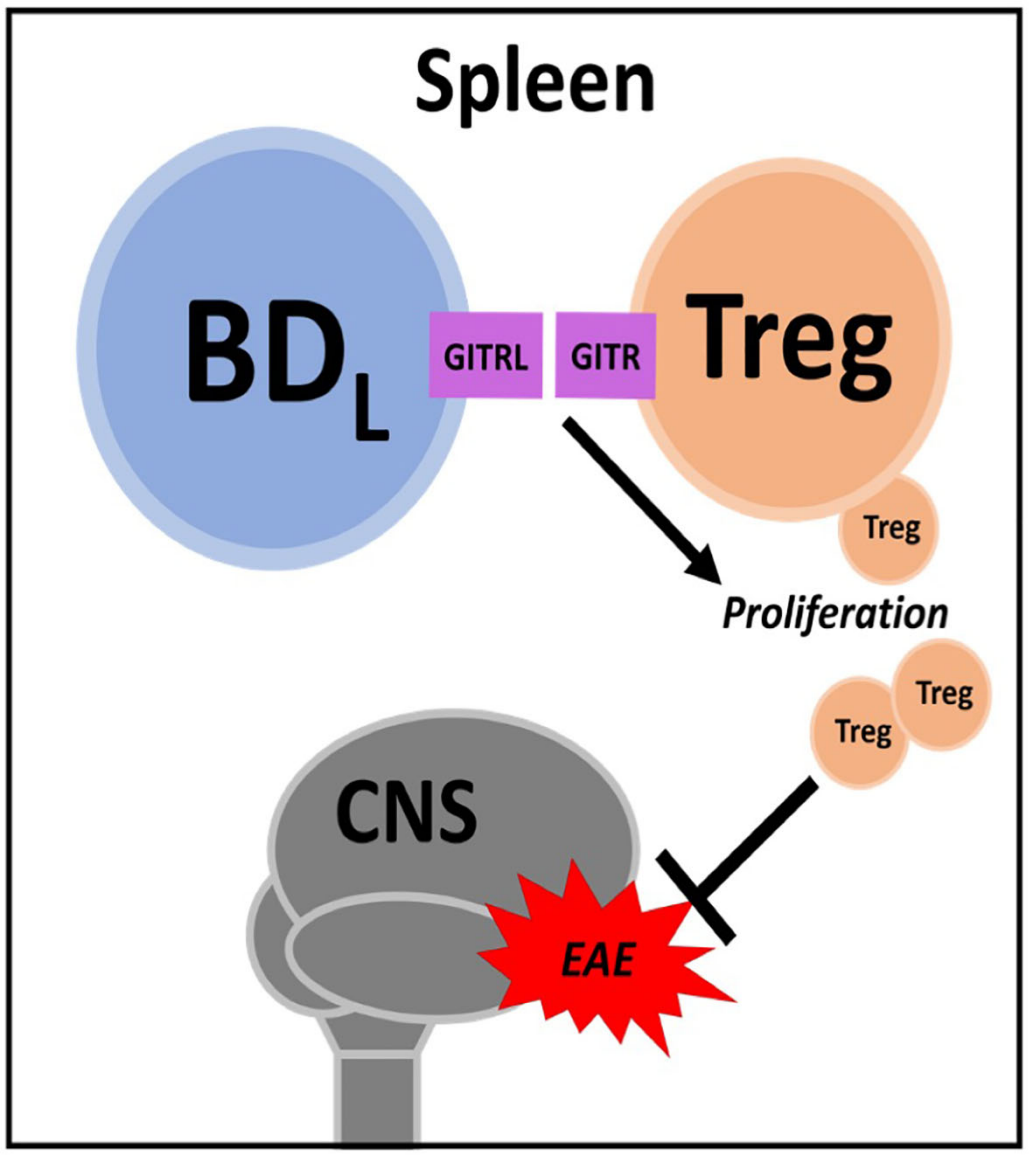
BD_L_. In the spleen BD_L_ promote regulatory T cell (Treg) proliferation through GITR-GITRL interactions. Treg attenuate experimental autoimmune encephalomyelitis (EAE) severity.

While the term “Breg” denotes a unique B cell subset, regulatory activity can be found in virtually all B cell subsets (25–28). Thus, Breg describes the general capacity for B cells to dampen immune responses but does not detail their exact nature, i.e., phenotype, mechanism of regulation or functional context. Many recent reviews summarize a multitude of mechanisms by which B cells are anti-inflammatory. For simplicity, these mechanisms are often divided into IL-10-dependent and -independent activities (10, 26, 28, 29). We support the concept that while all B cell subsets have contextual regulatory capacity, some subsets are inherently better suited than others. Because of this complexity, characterization of specific B cell subsets that exert regulatory activities has been incomplete and inconsistent across model systems and between research groups. Thus, there is a need for a definitive characterization strategy to assess novel regulatory B cell subsets. Here, we will review the history of B cell immune regulation research with focus on the discovery and testing of B cell subsets whose phenotype and mechanism of action have been clearly delineated. These include IL-10 producing plasmablasts/plasma cells (30–32) (**Figure 1**) and B cell IgD low (BD_L_) that interact with and induce proliferation of CD4^+^Foxp3^+^ T regulatory cells (Treg) (33) (**Figure 2**). In each of the four articles highlighted, negative immune regulation was assessed using the animal model of multiple sclerosis (MS), experimental autoimmune encephalomyelitis (EAE), and measured as the level of recovery from the signs of disease. Although B cell negative immune regulation has been reported in a variety of human disease models, for brevity and continuity, we are limiting this review to MS and its animal model EAE.

## The Discovery of B Cell Negative Immune Regulation

The discovery of a regulatory role for B cells in autoimmunity, ironically, was a serendipitous finding that was the outcome of a study in the laboratory of Dr. Charles A. Janeway, Jr. to determine whether B cell antigen presentation of peptides could induce CD4 T cell priming. This question was asked using EAE, which was well suited to address the question because of its dependence upon CD4 T cell priming to a self-antigen (34). EAE was induced in B10.PL (H-2^u^) mice by immunization with the myelin basic protein (MBP) immunodominant peptide Ac_1-11_ emulsified in complete Freund’s adjuvant accompanied by two doses of pertussis toxin (day 1 & 2) (35). This model was chosen because it was the first peptide active immunization model of EAE (36). To address the original question, B10.PL mice rendered deficient of mature B cells by disruption of the IgM heavy chain (B10.PLμMT) (37) were immunized with Ac_1-11_ (35). Now as part of scientific history, we showed that μMT mice not only succumbed to EAE, but failed to recover from the signs of EAE (35). This later finding is an advantage of using B10.PL mice because WT mice spontaneously recover from EAE. Although this result did not answer the original question of whether B cells can take up and present peptide antigens to T cells, it did indicate that B cell antigen presentation was not required for EAE induction. The observation that the mice did not recover from EAE provided the first evidence that B cells play a regulatory role in autoimmune responses. Remarkably, this finding was obtained prior to the identification of CD4^+^Foxp3^+^ Treg. The question then became, through what mechanisms do B cells facilitate resolution of EAE or other inflammatory conditions?

## IL-10/IL-35-Secreting Plasmablasts/Plasma Cells

IL-10 is a strong anti-inflammatory cytokine, which can potently suppress antigen presentation, among other functions, leading to attenuated immune responses (38, 39). IL-10-deficient mice develop a severe, nonrecovery EAE phenotype much like B cell-deficient animals (40, 41). Fillatreau and colleagues, in 2002, reproduced our findings that B cells are required for EAE recovery in C567BL/6 mice using the myelin oligodendrocyte glycoprotein (MOG) 35-55 peptide immunization model (42). Mechanistically, they were the first to demonstrate a role for B cell production of IL-10 in EAE recovery (42). This was accomplished utilizing IL-10-deficient mice and a combination of mixed BM chimeras and B cell transfers. Since this landmark finding, a multitude of subsequent studies have assessed B cell production of IL-10 through methods such as ELISA and ELISPOT, flow cytometry, fluorescent reporters and quantitative PCR (43). IL-10 production was commonly assessed following potent antigen non-specific *in vitro* B cell stimulation (44). While many, if not all, B cell subsets can produce IL-10 after *in vitro* stimulation, concurrent secretion of pro-inflammatory cytokines (IL-6, TNF-α) has been reported, suggesting that *in vitro* activation is not sufficient to assess true regulatory activity *via* IL-10 (25, 45). Indeed, B10 B cells named for their production of IL-10, are not a specific subset of B cells, but defined by IL-10 production, regardless of whether the IL-10 is regulatory in a disease context (28). Thus, measuring IL-10 production alone is not sufficient to define/phenotype B cell subsets with regulatory activity. The later requires mechanistic studies utilizing well-defined and/or purified B cell subsets, and as shown by Fillatreau and colleagues (42), the utilization of IL-10-deficient B cells.

Once it was recognized that B cell production of IL-10 was anti-inflammatory in EAE, the search commenced to identify the phenotype of the regulatory B cell. This was a daunting task given that all B cell subsets have the capacity to produce IL-10. In particular, B cells produce copious amounts of IL-10 following TLR stimulation (44, 46–49). Interestingly, CFA used to induce EAE has numerous TLR ligands and B cells stimulated *via* TLR ligands were shown to suppress EAE (44, 46). It took until 2014 for the identification of plasmablasts/plasma cells as the major source of B cell-derived IL-10 during EAE (30). In the first of two articles, Fillatreau and colleagues, used both EAE and *Salmonella* infection along with BM chimeras utilizing knockout (KO) mice, to demonstrate that IL-35 production was also a critical B cell-derived anti-inflammatory cytokine (30). IL-35 as a potential B cell regulator was identified *via* a microarray approach utilizing B cells expressing IL-10 as detected using IL-10 reporter mice. They went on to show that splenic plasma cells were the major contributors of IL-10 and IL-35. Here, we want to emphasize that thorough cell surface phenotyping (CD138^hi^TACI^+^CXCR4^+^CD1d^int^Tim1^int^) combined with intranuclear detection of the plasma cell transcription factor Blimp were utilized (30). This was the first time a “definitive” B cell phenotype was identified exhibiting regulatory activity *via* secretion of IL-10/IL-35 (**Figure 1**). IL-10/IL-35 production was used as the starting point to identify the B cells, not as the endpoint.

In the same year, Matsumoto and colleagues also reported that plasmablasts were the primary producers of IL-10 in EAE (31). IL-10 reporter mice (Venus) were used as the strategy to identify IL-10 producing B cells, which were highly prevalent in the draining lymph nodes, but not in the spleen or spinal cord on day 14 of EAE (~peak of disease). Using cell surface phenotyping and intranuclear Blimp-1 staining, CD138^+^ plasmasblasts/plasma cells were identified as the major IL-10 secreting cells during EAE. Confirmation that the plasmablasts/plasma cells were negative regulatory in EAE came from studies utilizing Blimp-1 B cell conditional KO mice. Additional studies indicated that lymph node, but not splenic plasmablasts/plasma cells were responsible for the negative regulation. To resolve the splenic (30) versus lymph node origin of the regulatory plasmablasts (31), adoptive transfer experiments were conducted, which ultimately demonstrated that the negative immune regulation occured in the lymph node independent of the germinal center (i.e., plasmablasts). In retrospect, the lymph node as the location of B cell-derived IL-10 immune regulation makes intuitive sense, due to IL-10s’ potent ability to suppress antigen presentation. MOG_35-55_ EAE is induced by immunization driving T cell priming due to delivery of the peptide to the draining lymph node *via* dendritic cells. IL-10 has been reported to negatively regulate the upregulation of MHC class II, co-stimulatory molecules and cytokines important for CD4 T cell priming (50, 51). Indeed, *in vitro* studies suggested that dendritic cells were the target of the plasmablast-derived IL-10 (31). TLR2, 4 and/or 9 agonists in the CFA likely induced the IL-10 production by the plasmablasts/plasma cells (30, 31). Here, again, IL-10 production was the starting point and the implementation of multiple strategies were utilized to definitively identify the B cell subset that produced the regulatory IL-10 (**Figure 1**).

In addition to autoimmunity, B cell activation and differentiation into antibody-secreting plasma cells is also associated with IL-10 competency and immunosuppressive activity in cancer (31, 52–54). While plasmablast/plasma cell characterization has narrowed the phenotype for IL-10-secreting B cells, several plasma cell populations exist between the BM, secondary lymphoid organs (spleen/lymph nodes) and mucosal sites (i.e., gut) (55–60). Of importance, not all PC can produce IL-10, such as IgG-secreting plasma cells (53). Interestingly, microbial sensing through TLR (54) and the presence of gut commensals (61) seems required for effective plasmablast/plasma cell immunosuppression (30, 31). The gut microbiome and its role in MS has been investigated using models like germ-free, single microbe monocolonized and specific pathogen-free animals in tandem with EAE and by sequencing microbiomes from human patients (62–64). While a definitive MS microbiome is yet unclear, these cumulative studies have identified microbes associated with disease activity as well as quiescence and elucidated further mechanisms through which the gut microbiome impacts immune regulation (65–67). While a multitude of mechanisms exist, IgA production in the gut has emerged as an important regulator of the composition of the gut microbiome. IgA is the most abundant Ig in the body. In human adults, around 5 mg is secreted at mucosal surfaces per day (68, 69). IgA is produced by gut plasma cells residing in the lamina propria of the small and large intestine and is secreted as a dimer joined covalently by the J-chain protein which acquires an additional secretory component during translocation into the gut lumen *via* the epithelium (70–72) (**Figure 1**). In the gut, IgA canonically targets microbes through antigen-specific binding but can also participate in non-specific binding *via* glycan-, J-chain-, and secretory component-driven adherence (72–74). Through these combined mechanisms, IgA facilitates clearance of potentially pathogenic microbes while allowing beneficial commensals to thrive (75, 76).

Rojas and colleagues, investigated whether there was a link between gut IgA, IL-10 and EAE penetrance (32). They identified gut-derived IgA-secreting plasma cells as a potent source of IL-10 and that these cells ameliorated EAE by trafficking from the gut to the brain. In their work, IgA PC were characterized by multiple techniques including fluorescent reporters with parabiosis tracking, flow cytometry and microscopy and were functionally assessed *via* ELISPOT and adoptive cell transfer analysis. These plasma cells were shown to be IgA- and IL-10-secreting, expressed Blimp-1, had low levels of canonical B cell markers (B220) and reacted with gut-derived antigens *in vitro* (32) (**Figure 1**).

While each of the three highlighted studies discussed above were comprehensively and elegantly conducted using state-of-the-art techniques, one can ask how does induction of EAE with immunization utilizing a potent TLR stimulating adjuvant (CFA) have relevance to human MS? The nature of this question is relevant to most animal models of human disease. Nevertheless, interestingly, all FDA approved therapies for MS have shown efficacy in the EAE model of MS. Although little is known regarding the role of IL-35 in MS, it has been shown to be elevated in the serum of treated patients as compared to the control group (77). A second study found that IL-35 was reduced in relapsing-remitting MS patients as compared to healthy controls (78). A third study reported that IL-35 was higher in MS patients as compared to healthy controls (79). In a fourth study, MS patients infected with helminths had higher levels of IL-35-producing B cells in the CSF, which correlated with reduced MRI lesions, as compared to uninfected patients (80). *In vitro* studies, showed that IL-35 could drive IL-35 and IL-10 production by CD19^+^ B cells and induce a Treg-like phenotype (80). While these studies are interesting, the lack of definitive B cell phenotyping limits impact. Taken together, the collective IL-35 data indicate that much remains unknown regarding the source of IL-35 in MS and its mechanism of action. Human relevance investigated by Matsumoto and colleagues utilized an *in vitro* culture system whereby healthy human peripheral blood B cells were stimulated through TLR9 with CpG along with IFN-α/IFN-β to induce IL-10 production (31). It was found that of the various plasmablast populations generated, those derived from naïve B cells, with a CD27^int^CD38^+^ phenotype, expressed the highest level of IL-10 and remained IgM^+^. Whether these cells are regulatory in MS is not known. In the Rojas study, MS patients were utilized to demonstrate that IgA bound bacteria was reduced during active disease (32). The presence of IgA-producing plasma cells in the MS brain has since been confirmed (81). While the gut microbiota is closely associated with IgA-secreting plasma cells, the signals required for IL-10 secretion and migration to sites of autoimmune inflammation require further exploration.

In summary, although plasmablasts/plasma cells identified by Blimp-1 expression were shown to be the regulatory B cell in EAE, each study identified a different mechanism. In the spleen, they regulated *via* IL-35, *via* an unknown mechanism (30) (**Figure 1**). In the lymph node, IL-10 was the primary immunosuppressive cytokine that likely targeted dendritic cell antigen presentation (31) (**Figure 1**). While in the gut, IgA plasma cells trafficked to the CNS and suppressed *via* IL-10 (32) (**Figure 1**). While the IL-10 target in the CNS was not investigated, it was shown that overexpression of IL-10 in the CNS *via* transfer of immortalized fibroblasts producing IL-10 or an adenovirus expressing IL-10 into the CNS potently attenuated EAE severity (82, 83). These collective studies illustrate the complexity of B cell regulation within just the plasmablast/plasma cell subset, which itself is a complex group of cells with differential production of Ig isotypes, anatomical location, cytokine profiles and longevity.

## Maintenance of Treg Homeostasis by BD_L_

As discussed above, we were the first to demonstrate a negative regulatory role for B cells in promoting recovery from EAE (35). This seminal study was conducted using the active immunization model utilizing CFA. To avoid bystander effects of CFA (i.e., TLR agonism), we first confirmed that B10.PLμMT mice did not recover from EAE induced by the passive transfer of MBP-specific encephalitogenic CD4^+^ T cells generated from MBP-TCR transgenic mice (84, 85). Interestingly, in this study utilizing BM chimeras, we did investigate IL-10 expression, but in the context of the recently described CD4^+^Foxp3^+^ Treg, not B cells (85). In a follow up study, we utilized anti-CD20 B cell depletion as an alternative to genetic disruption (μMT) to render mice B cell deficient, to further support our findings that B cells are required for EAE recovery (86). In this study, we moved from a BM chimera approach to B cell adoptive transfer and showed that neither IL-10 nor MHC class II were required for B cells to drive EAE recovery. Keeping with our previous findings, we investigated a role for Treg and found that B cells induced their proliferation in a glucocorticoid-induced tumor necrosis factor receptor ligand (GITRL)-dependent manner (**Figure 2**). This mechanism was supported by findings that mice deficient in B cells (either μMT or anti-CD20 depleted) had a significant reduction in splenic Treg and that B cell adoptive transfer into μMT mice restored Treg numbers and recovery from EAE. Antibody blocking of GITRL prior to B cell adoptive transfer, attenuated Treg expansion and EAE recovery.

By utilizing two different EAE models (active & passive) and two means to render mice deficient in B cells (genetic & antibody depletion), we were confident that B cells were required for recovery from EAE (85, 86). We then required a strategy by which to identify and subsequently purify the regulatory B cell subset. Unlike IL-10, we could not utilize GITRL due to its very low expression on B cells (86). We did perform some targeted adoptive transfers using FACS purified B cell subsets, but found that strategy too laborious, costly and unfocused. Thus, we chose anti-CD20 partial B cell depletion. By swabbing the IgG_2a_ heavy chain of the totally B cell depleting anti-CD20 to IgG_1_, B cell depletion efficiency was reduced (33, 87). We would like to thank Biogen Idec for generously providing these antibodies. Anti-CD20 IgG_1_ depleted the majority of follicular B cells while sparing marginal zone B cells (33). Thus, we were surprised that the B cell regulatory activity resided within the few follicular B cells that were not depleted (33). We then employed extensive flow cytometry cell surface phenotyping to determine that the non-depleted follicular B cells were enriched for IgD^low^ expressing B cells. Using FACS purified follicular IgD^low^ B cells, we showed that upon transfer into μMT mice, they drove Treg proliferation and EAE recovery. Importantly, neither follicular IgD^hi^ nor marginal zone B cells exhibited regulatory activity. Given that only follicular IgD^low^ B cells were regulatory in the context of our EAE model in conjunction with RNAseq studies, this evidence supported that we had identified a new B cell subset and named them B cell IgD low (BD_L_). Several strategies were utilized to determine that BD_L_ are not part of the marginal zone B cell lineage. Developmental studies showed that BD_L_ emerge in the spleen after the transitional 2 subset, similar to follicular B cells. However, how BD_L_ fit into follicular B cell development is not known. A more comprehensive review of how BD_L_ were discovered and the assays used to identify then were recently published (88, 89).

We too, were interested in whether our findings were relevant to humans. In that regard, we found that BD_L_ activity existed in IgD^low^ B cells in both human spleen and peripheral blood, as determined using an *in vitro* Treg expansion assay (33). However, a definitive human BD_L_ phenotype was not identified nor were studies in MS performed. Thus, much remains to be discovered regarding B cell biology, particularly in the context of disease. In summary, by utilizing a variety of experimental approaches combined with comprehensive cell surface phenotyping and mechanistic studies, like IL-10 secreting plasmablasts/plasma cells, we have successfully identified a definitive B cell subset with regulatory activity (33, 88).

## B Cell Phenotyping to Identify Regulatory B Cell Subsets

**Figure 3:**
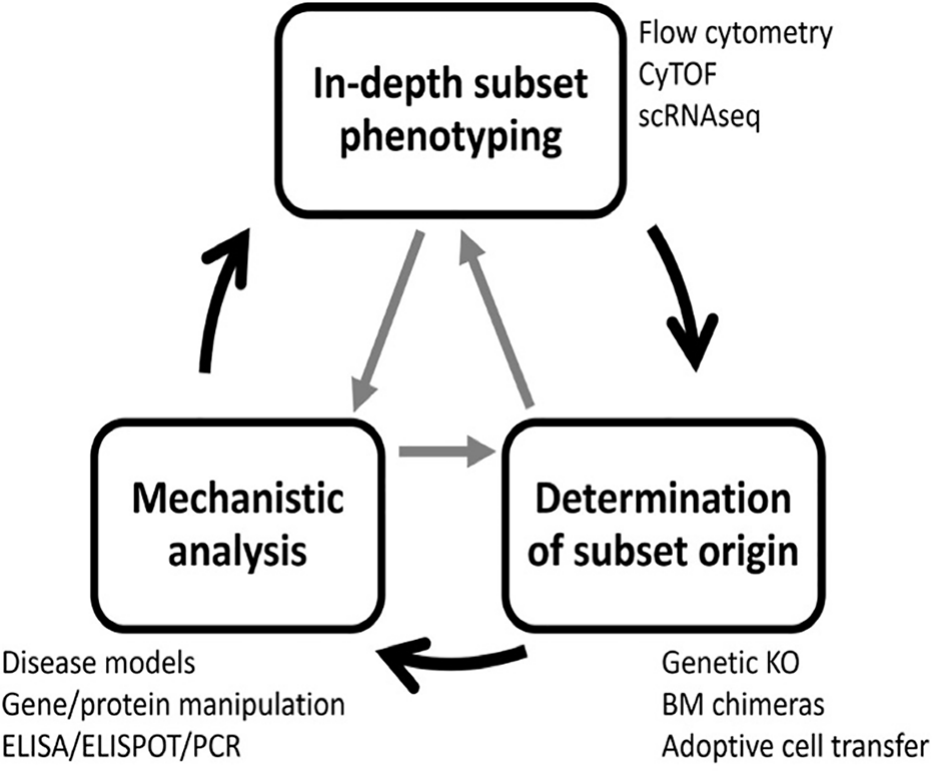
A three-step process to identify regulatory B cell subsets. Complete characterization of novel regulatory B cell subsets requires three steps: subset phenotyping, lineage determination, and functional analysis. To accomplish this goal, many experimental techniques are required. The process is often iterative, and steps can be completed in any order and revisited as new information is gained.

Previous research has uncovered many B cell subsets with regulatory potential that utilize a large cadre of regulatory mechanisms (10, 26). This highlights the complexity and challenges in targeting these populations for in-depth testing and therapeutic development. Here, we describe a strategy for identifying regulatory B cell subsets using gold-standard and state-of-the-art methodology that has three steps: 1) in-depth B cell subset phenotyping, 2) determination of B cell subset origin and 3) functional/mechanistic analysis. These steps do not need to be performed in a specific order, and the process of definitively characterizing a novel subset is often iterative and cyclic in nature (**Figure 3**).

### In-Depth B Cell Subset Phenotyping

**Figure 4:**
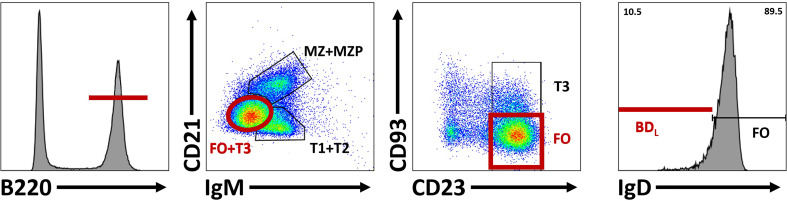
Differentiation of BD_L_ from other splenic B cell subsets. To identify BD_L_, B220^+^ cells are selected and subsequent comparison of lgM and CD21 subdivides B cells into three groups based on maturity: marginal zone (MZ) and marginal zone precursors (MZP), transitional 1 (TI) and T2 cell and T3 and follicular (FO) B cells. CD23 and CD93 are subsequently used to identify FO B cells. Within the FO B cell subset, BD_L_ are selected for their low/negative expression of lgD.

**Figure 5:**
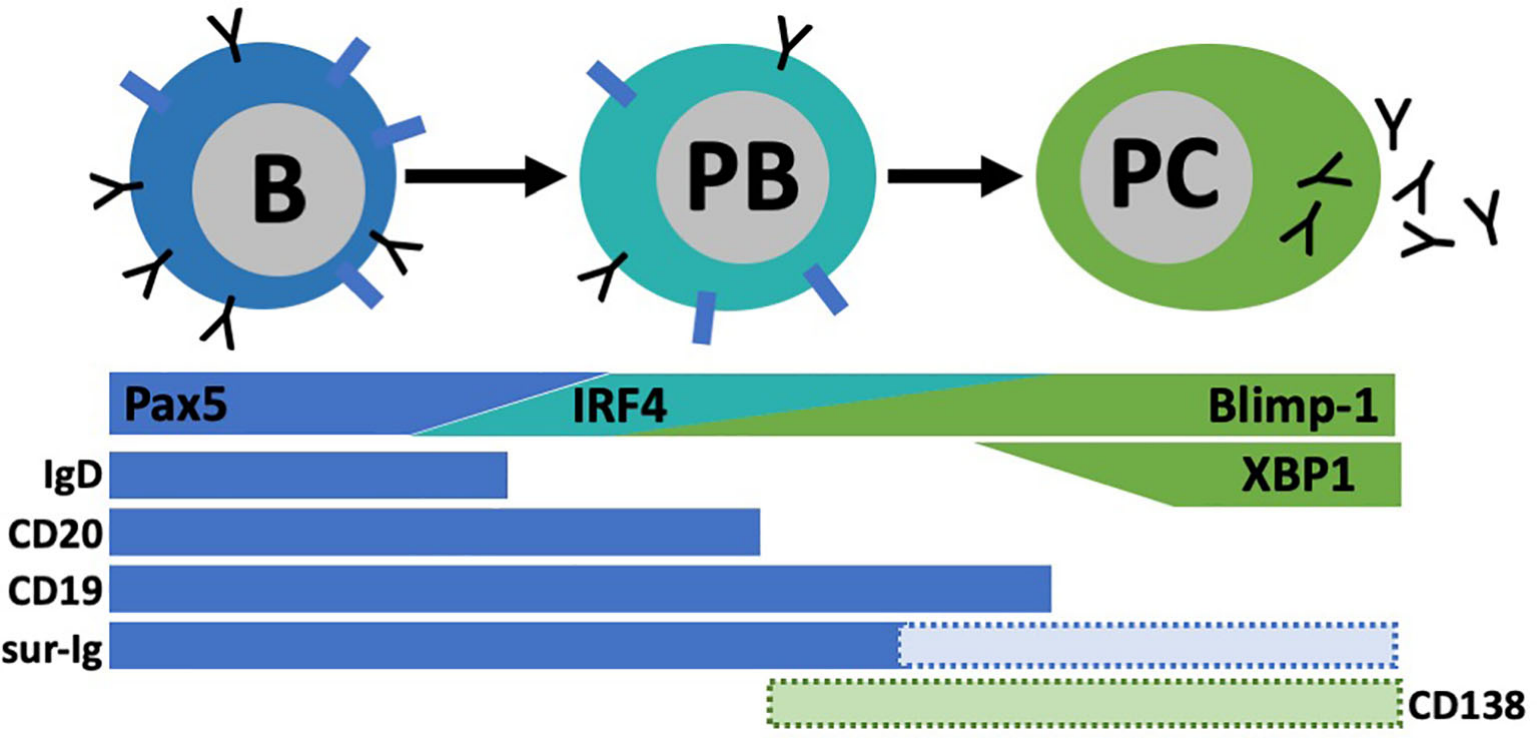
Plasmablast (PB)/pIasma cell (PC) differentiation. B cells are characterized by their high expression of transcription factor Pax5 and surface markers lgD, CD20, CD19, and surface lg (sur-lg). When B cells differentiate into PC, they transverse through a PB stage known for its high proliferative capacity. As PC maturation continues, Pax5 expression is lost and other transcriptional programs (IRF4, Blimp-I, XBPI) are upregulated. Concurrently, most B cell surface proteins are lost. Some subsets of PB and PC have expression of CD138 among other markers, but not all express these.

For in-depth B cell subset phenotyping, flow cytometry is a powerful tool that allows for comparative analysis of many cell surface and intracellular/intranuclear markers combined. However, conventional flow cytometry is limited in breath due to an upper limit of 12-14 colors (markers) with a conventional flow cytometer equipped with four lasers. If the flow cytometer also has a UV laser, 20 colors are possible. However, realistically, most laboratories have a upper limit of 6-8 colors. In our studies, identification of BD_L_ required six colors that includes B220 or CD19, IgM, IgD, CD21, CD23 and CD93 (33) (**Figure 4**). In addition, this combination of cell surface markers can be used to identify splenic transitional subsets, the marginal zone lineage and follicular B cells (**Figure 4**). In unpublished studies, we have now identified an additional BD_L_ marker making our panels seven colors. We also routinely phenotype Treg (CD4^+^Foxp4^+^) and if done in combination with B cell phenotyping would be nine colors. If B cells are enzymatically obtained from tissues the hematopoietic marker CD45 along with a live/dead stain need to be included. Determining IL-10 expression requires either a reporter or intracellular staining. Similarly complex, delineation between mouse plasmablasts and plasma cells from other B cell subsets requires both cell surface phenotyping, intracellular (IL-10/IL-35) and intranuclear transcription factor identification (i.e., Blimp-1, IRF4), because no cell surface markers alone identify these subsets (**Figure 5**). Surface markers including CD19^low/neg^, B220^low^, CD20^-^, CD38^+^, CD44^hi^, CD27^+^, CD138^+^, TACI^+^, CXCR4^+^, Tim1^+^, cell surface Ig^low/-^ are used in addition to transcription factor identification to further characterize subsets of plasmablast/plasma cells (**Figure 5**). One major hurdle in identifying BD_L_ and plasmablasts/plasma cells is that they are rare, making their purification for downstream analysis challenging. Because transcription factor staining for flow cytometry kills target cells, Blimp-1 reporter animals are necessary to FACS purify plasmablasts/plasma cells (90). Additionally, IgM-targeting can activate B cells so care must be taken when purifying cell subsets using this marker. Other markers associated with B cell regulation to consider are CD5, CD1d, PD-L1 and FasL (10, 26). In mouse studies, it is important to analyze several lymphoid tissues to determine if the novel B cell regulatory subsets are sequestered to a portion of the body or more generally found in multiple tissue types.

B subset markers in mouse and human are relatively similar, but differences do exist. CD10 is a human B cell marker not used in mice and CD38 is used more often in humans than mice (60). Human tissues beyond peripheral blood can be difficult to obtain and B cells in circulation do not allow the analysis of specialized B cells subsets such as marginal zone B cells, germinal center B cells, long-lived plasma cells or mucosal B cell subsets. Human B cell subsets are also infinitely more complex than mice due to exposure to environmental and microorganism-derived antigens leading to isotype class switching and memory and plasma cell generation. Here, BD_L_ will be used to illustrate human B cell complexity. Although, we examined a number of B cell markers to narrow the BD_L_ phenotype in humans (unpublished observations), in the end, the only reliable phenotype was CD19^+^ (or equivalent B cell marker) and IgD^low^. In addition to BD_L_, the CD19^+^IgD^low/-^ phenotype can include: 1) isotype class switched effector B cells, 2) isotype class switched memory B cells, 3) IgM memory B cells, 4) circulating transitional B cell subsets and 5) circulating marginal zone B cells. It is unlikely that flow cytometry alone could differentiate between the various IgD^low/-^ human B cell subsets. Thus, newer technologies may need to be applied. These include imaging mass cytometry (CyTOF), which is a mass spectrometry approach that utilizes metal conjugated antibodies to identify ~50 parameters/cells with minimal overlap in the metal signals (91). Primary disadvantages include its lack of mainstream use, slow flow rate and destruction of the analyzed cells. Spectral flow cytometry is a newer technology that also has the advantage of minimal overlap of signals. While similar to conventional flow cytometry, its fluorescent emission spectrum is captured by detectors across several defined wavelength ranges. In essence, every molecule’s fluorescent spectrum is recorded as a spectral signature that can be discriminated from other fluorescent signatures (92). Spectral flow cytometers are now commercially available and are slowly being incorporated into basic research studies. Finally, single cell RNA sequencing (scRNAseq) has become a strategy of choice to identify rare subsets of immune cells (93). Detection of cell surface proteins *via* antibodies (i.e., TotalSeq™) can be used alongside scRNAseq strategies (i.e., 10X Genomices) to analyze the transcriptional profiles of pre-marked immune cell subsets (94). In addition, full-length BCR sequences can also be obtained (95). The best platform to choose is dependent upon the experimental question. 10X Genomics allows the analysis of high numbers of individual cells (thousands) and can be combined with cell surface identification. However, its primary weakness is that sequence read depth is limited to high copy number transcripts, so unique markers expressed at low levels may be washed out during analysis. In comparison, both the Fluidym and Takara systems can analyze sequences with low copy number transcripts, but fewer cells can be analyzed per run (hundreds) (93, 96, 97).

### Determination of B Cell Subset Origin

B cell phenotyping can provide strong evidence of the lineage from which a particular B cell subset derives. However, additional developmental studies should be performed. In comparison with conventional B2 B cells from the BM, B1 B cells are derived from the yolk sac/fetal liver, thus they can be eliminated with BM transplantation (98). Marginal zone B cells require Notch-2 signaling for development and any disruption in that pathway prevents their differentiation (99). Plasmablast/plasma cell differentiation can be prevented by loss of Blimp-1 expression (100). The loss of XBP-1 in plasmablasts/plasma cells can be used interrogate relevance of Ig secretion with other mechanisms (101). Germinal center B cells require BCL6 and its deficiency leads to truncation of the germinal center response (102). The transcription factor ABF-1 prevents plasma cells differentiation, without disrupting memory (103). Bach2 was recently shown to be essential for the transition from germinal center B cells to memory B cells (104). Undoubtably, other transcription factors can also be targeted to disrupt the development of specific B cell subsets. The developmental timing/kinetics of any B2 subset can be tracked after BM transplantation or sublethal irradiation (33). In addition, adoptive transfer is also an effective strategy for monitoring B cell subsets, especially when used in tandem with congenic markers (CD45.1/2) (33). For instance, the adoptive transfer of transitional 2 cells leads to the emergence of both the follicular and marginal zone lineages, as well as BD_L_ (33). When used together, these KO and cell reconstitution strategies can experimentally interrogate the lineage of regulatory B cell activity.

### Functional/Mechanistic Analysis

Beyond identifying definitive markers of regulatory B cells subsets and where they come from, characterization of their functional capacity is equally important. Functional capacity includes 1) the mechanism through which the regulatory B cell yields an altered immune response and 2) the context in which this is applicable (type of disease, target cells). As evidenced through the story of B cells in EAE, not every regulatory B cell activity is relevant in all model systems and potentially multiple B cell subsets can be regulatory through different mechanisms at the same time. Thus, choosing and accurately describing the disease context is important for characterizing the regulatory B cell subset. Mechanisms of regulatory B cells can be investigated using many of the approaches discussed above where cellular activities (i.e.; IL-10/IL-35 secretion) are altered by gene/transcript-targeted deletion/overexpression or protein inhibition/stimulation with drug compounds. Genetic animal strains (including B cell-conditional varieties), BM chimeras, adoptive cell transfers and *in vitro* co-culture systems are used in tandem with these approaches to minimize confounding effects of gene manipulation on other, non-B cells. Further methods for investigating regulatory B cell mechanisms include measuring cytokines and effector molecule production *via* reporters (i.e.; IL-10, Blimp-1), ELISA, ELISPOT, quantitative PCR and RNAseq. While conducting these analyses, it is important to keep in mind the purity of tested cell subsets, this is where in-depth B cell phenotyping can aid in and strengthen the interpretation of results.

## Concluding Remarks

To ensure the accurate identification of novel B cell subsets with regulatory activity and their mechanism, consistent and comprehensive phenotyping along with mechanistic studies are necessary. The four laboratories, including our own, whose work was discussed in this review were chosen because they all utilized multiple strategies to identify a definitive B cell subset whose mechanism of action was confirmed using multiple mechanistic approaches. These included KO mice, BM chimeras, genetic approaches and adoptive cell transfer, among others. Although each story was years in the making, cumulatively, they provide a path by which others can follow to discover novel regulatory B cell subsets and mechanisms in the context of other diseases. Similar investigations have been made in models beyond MS/EAE that researchers interested in B cell regulation should explore. The identification of regulatory B cell subsets and their mechanism leads to the question of how they can be exploited for the treatment of anti-inflammatory diseases. The main hurdle with utilizing B cells as an adoptive cell therapy is that they do not self-renew, like their T cell counterparts. Thus, longevity must be addressed. In the context of IL-10, adoptive transfer of long-lived plasma cells that produce IL-10 is being actively explored. We are actively exploring how BD_L_ can be utilized as an adoptive cell therapy to increase Treg numbers in autoimmunity. These are exciting times for research in B cell regulation and given the complexity of the entire B cell lineage new B cell subsets and regulatory mechanisms are surely to be discovered.

## Author Contributions

BD and SN both wrote and edited the article. All authors contributed to the article and approved the submitted version.

## Funding

This work was supported by the NIH grants R01AI160244-01 and R21AI145323 (BND), the National Multiple Sclerosis Society grant RG-1901-33315 (BD) and the Versiti Blood Research Institute Research Foundation.

## Conflict of Interest

The authors declare that the research was conducted in the absence of any commercial or financial relationships that could be construed as a potential conflict of interest.

## Publisher’s Note

All claims expressed in this article are solely those of the authors and do not necessarily represent those of their affiliated organizations, or those of the publisher, the editors and the reviewers. Any product that may be evaluated in this article, or claim that may be made by its manufacturer, is not guaranteed or endorsed by the publisher.
